# A Case of Oncocytic Adrenocortical Neoplasm of Borderline (Uncertain) Malignant Potential

**DOI:** 10.7759/cureus.638

**Published:** 2016-06-13

**Authors:** Mina Shenouda, Linda G Brown, Krista L Denning, Toni Pacioles

**Affiliations:** 1 Internal Medicine, Joan C. Edwards School of Medicine at Marshall University; 2 Pathology Department, Joan C. Edwards School of Medicine at Marshall University; 3 Hematology/Oncology Department, Edwards Comprehensive Cancer Center, Joan C. Edwards School of Medicine at Marshall University

**Keywords:** oncocytoma, adrenocortical neoplasm, borderline malignant potential, papillary thyroid carcinoma

## Abstract

Oncocytic neoplasms are tumors composed predominantly or exclusively of oncocytes (large polygonal cells with granular eosinophilic cytoplasm due to abnormal mitochondrial accumulation). These tumors are frequently reported in the thyroid, kidneys, and salivary glands. However, they are distinctly rare in the adrenal cortex. Oncocytic adrenocortical neoplasms (OAN) are classified regarding their biological behavior by their histological features according to the Lin-Weiss-Bisceglia system (LWB). Here, we report a case of OAN of borderline or uncertain malignant potential (BMP) with subsequently identified papillary thyroid carcinoma (PTC). A 34-year-old female with a nine-month history of fatigue presented with chest pain. A right adrenal mass was incidentally found while ruling out pulmonary embolism. A CT-guided adrenal biopsy, although not routinely indicated, was performed and interpreted as malignant with no definitive origin. Hormonal workup was unremarkable. PET-scan showed hypermetabolic adrenal mass with peak standardized uptake value of 15, suspicious of malignancy. A hypermetabolic thyroid nodule was also identified, but there was no evidence of metastatic disease. The patient underwent adrenalectomy, and the initial pathology report was interpreted as atypical pink cell tumor. A second pathology report from another laboratory favored OAN based on the morphology and immunohistochemical staining. While the histologic criteria of malignancy were not met, the large tumor size makes it compatible with BMP according to LWB criteria. A follow-up thyroid ultrasound revealed a complex thyroid nodule. A total thyroidectomy was performed, and pathology was consistent with PTC. Of interest, PTC frequently shows an increase in mitochondrial content, which is characteristic of oncocytic tumors. This case illustrates that OAN, although rare, should be considered in the differential diagnosis of adrenal masses. When OAN is identified, it should be classified regarding its biological behavior as benign or malignant using the LWB system and, eventually, the reticulin algorithm of Duregon, et al. Oncocytoma can be confirmed ultrastructurally or by immunohistochemistry. Studying the gene mutations in patients presenting with oncocytic malignancies and other tumors that demonstrate mitochondrial proliferation as PTC might help to understand the role of mitochondrial proliferation in cancer development.

## Introduction

Adrenocortical neoplasms are the most frequent abnormalities of the adrenal cortex. They are found in about 1% of the general population, increasing with age to 6% in the elderly [1]. Oncocytic adrenocortical neoplasm (OAN) is a very unusual variant of adrenocortical tumors. Their description in the literature is limited to single case reports and small series; to date, roughly 150 cases have been reported in the literature [2-3].

OANs are tumors composed exclusively or predominantly of oncocytes: large polygonal cells with granular eosinophilic cytoplasm due to abnormal mitochondrial accumulation [4]. There is no single parameter to discriminate between benign and malignant OANs, and they are classified regarding their biological behavior by a combination of histological features according to the Lin-Weiss-Bisceglia system (LWB) [5].

Most OANs are benign, non-functioning, and detected incidentally with a median age at diagnosis of 46 years. OANs are more frequently found in females [6]. OAN with BMP appears to have a relatively benign clinical behavior. However, recurrence was reported four years after the resection of an OAN with BMP [7]. Also, recurrence has been described in patients with an adrenal oncocytic carcinoma up to seven years after the removal of an adrenal tumor [8]. Accordingly, these tumors require long-term follow-up and a thorough clinical, hormonal, and imaging evaluation.

This article reports a case of an oncocytic adrenocortical tumor of borderline or uncertain malignant potential (BMP) with subsequently identified papillary thyroid carcinoma (PTC). Of interest, PTC frequently shows an increase in mitochondrial content, which is characteristic of oncocytic tumors. To our knowledge, this is the first article to report a concurrent OAN and PTC.

## Case presentation

The patient is a 34-year-old female with no significant medical history, except for hypertension and tobacco smoking. She had an unremarkable family history and no history of radiation exposure. Her symptoms started with fatigue and weight gain of about 80 pounds over nine months for which she did not seek medical attention. She presented to the hospital with chest pain and shortness of breath. A CT of the chest was done and ruled out pulmonary embolism. However, it revealed a partially visualized large right adrenal mass. Further evaluation with a CT of the abdomen and pelvis demonstrated a large mass of the right adrenal gland measuring 11 x 10 x 6 cm in size. The radiologist reported that the size and inhomogeneous appearance of the mass were suspicious findings and a primary adrenal carcinoma or metastatic disease needed to be excluded. Informed patient consent was obtained for treatment.

A CT-guided core biopsy of the adrenal mass, although not routinely indicated, was performed and interpreted as malignant, likely consistent with renal cell carcinoma or adrenal cortical carcinoma by morphology and immunostaining. However, the negative paired box gene 8 (PAX8) and the ambiguity of where the lesion was located precluded a definitive diagnosis.

A biochemical workup was performed to rule out the presence of a primary functioning mass, such as Cushing's syndrome, aldosteronoma, and pheochromocytoma. It was found to be unremarkable, including aldosterone (1.5 ng/dL), metanephrines (< 10 pg/mL), renin (0.19 ng/mL/hr), ACTH (9.4 pg/mL), and serum cortisol, which was 3.3 ug/dl at baseline and 0.7 ug/dl after low-dose dexamethasone suppression tests.

**Figure 1:**
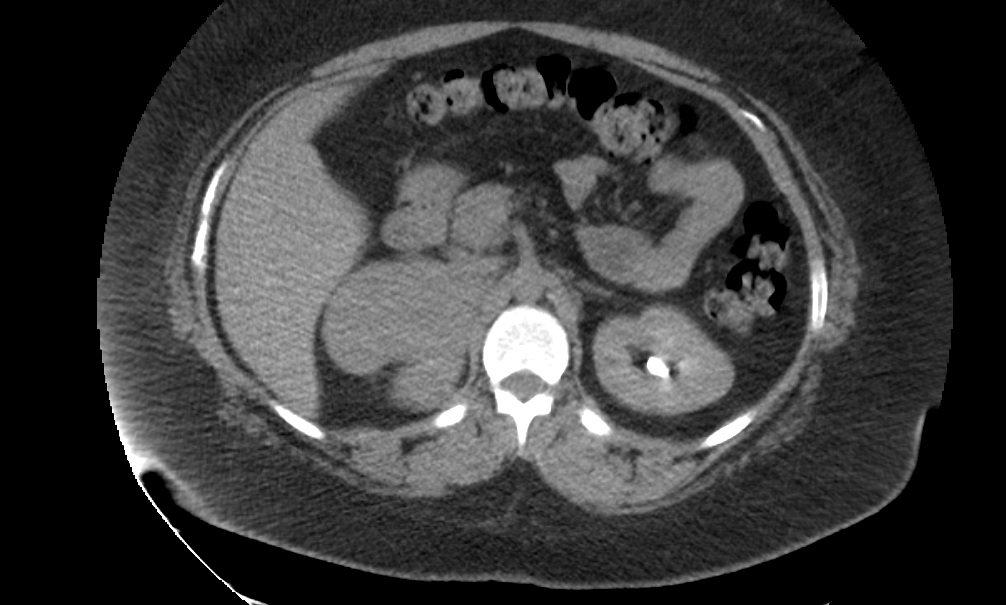
Preoperative CT of the abdomen and pelvis (axial view) showing multilobulated mass involving the right adrenal gland.

**Figure 2:**
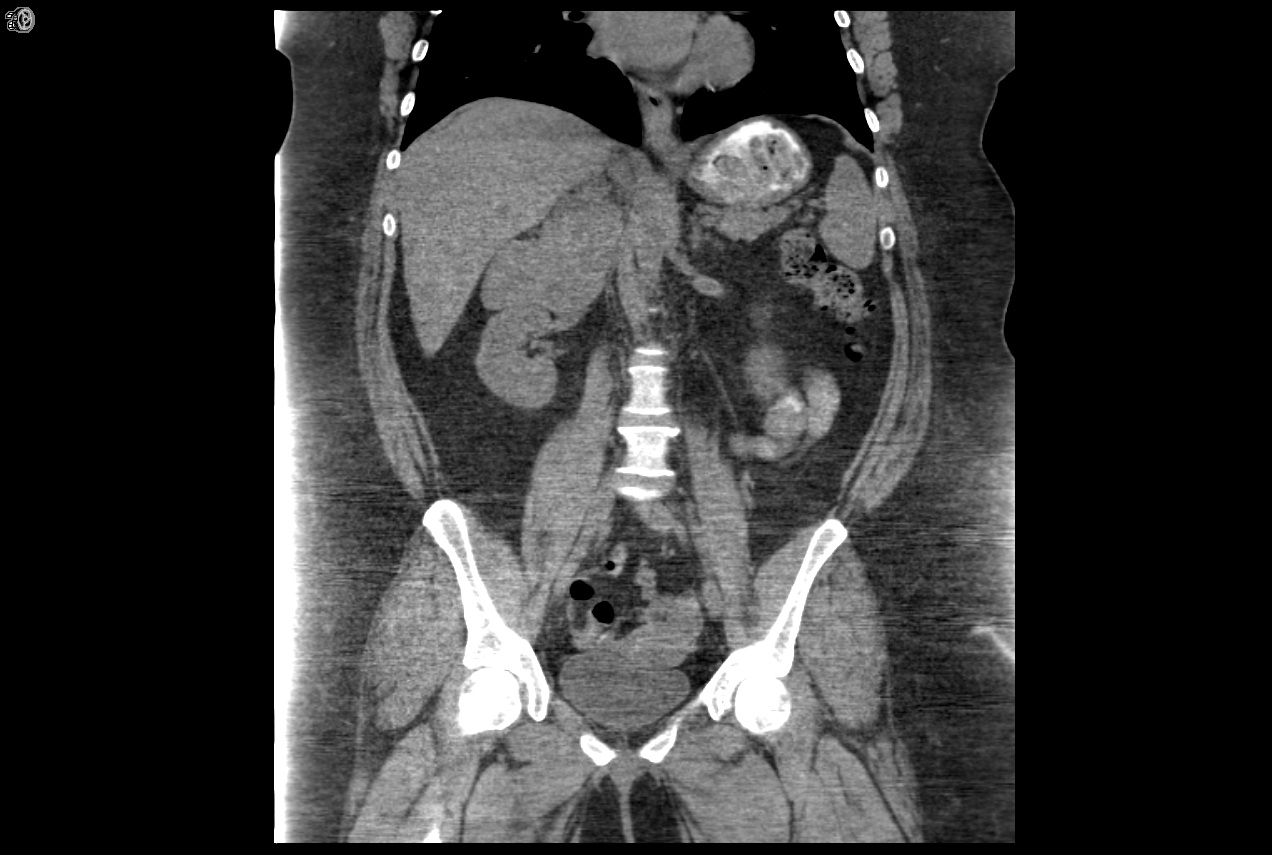
Preoperative CT of the abdomen and pelvis (coronal view) showing multilobulated mass involving the right adrenal gland.

**Figure 3:**
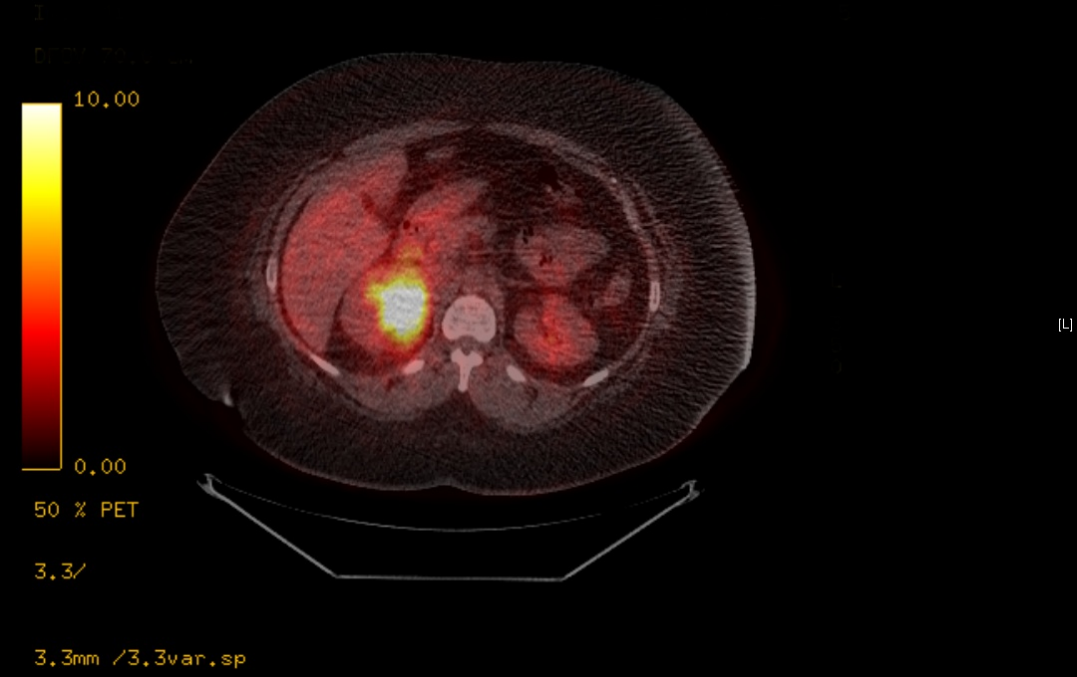
Preoperative PET-CT of the abdomen showing large right adrenal mass that is markedly hypermetabolic with peak SUV of 15.0.

A repeat CT of the abdomen performed at another facility showed a multilobulated mass with an inhomogeneous appearance involving the right adrenal gland measuring 8 x 8.3 cm with no evidence of metastatic disease (Figures 1-2). A PET-CT scan was performed and showed a markedly hypermetabolic right adrenal gland mass with a peak standardized uptake value (SUV) of 15, highly suspicious of malignancy with no evidence of metastatic disease (Figure 3). However, there was a markedly hypermetabolic isthmic thyroid nodule suspicious for thyroid carcinoma, and a follow-up thyroid ultrasound was recommended. A bone scan showed no evidence of bony metastatic disease.

The patient underwent a right adrenalectomy. The adrenal gland measured 12.7 x 9.4 x 4.8 cm and weighed 318 grams. On sectioning, a pink-red lesion was identified, which abutted multiple margins, and a rim of tissue consistent with normal adrenal gland was identified.

Surgical pathology reported that the tumor consisted of nests of cells with abundant eosinophilic cytoplasm. There was significant cytologic atypia, yet mitoses were not frequent, and no necrosis was seen. The differential diagnosis based on the H&E slide included renal cell carcinoma, adrenal cortical carcinoma, melanoma, paraganglioma, epithelioid GIST, hepatocellular carcinoma, perivascular epithelioid cell tumor (PEComa), gynecologic tumor, and alveolar soft part sarcoma. However, the following immune-histochemical staining pattern did not support any of these diagnoses: negative cytokeratin AE1 /AE3, PAX 8, inhibin, calretinin, S100, MART1, human melanoma black 45 (HMB-45), cathepsin K, ARG-1, Hep Par 1, TFE3, c-kit, and chromogranin. CAM 5.2 and vimentin showed staining on the periphery, but not throughout the lesion, such that they were also interpreted as negative. The final diagnosis was interpreted as atypical pink cell tumor.

**Figure 4:**
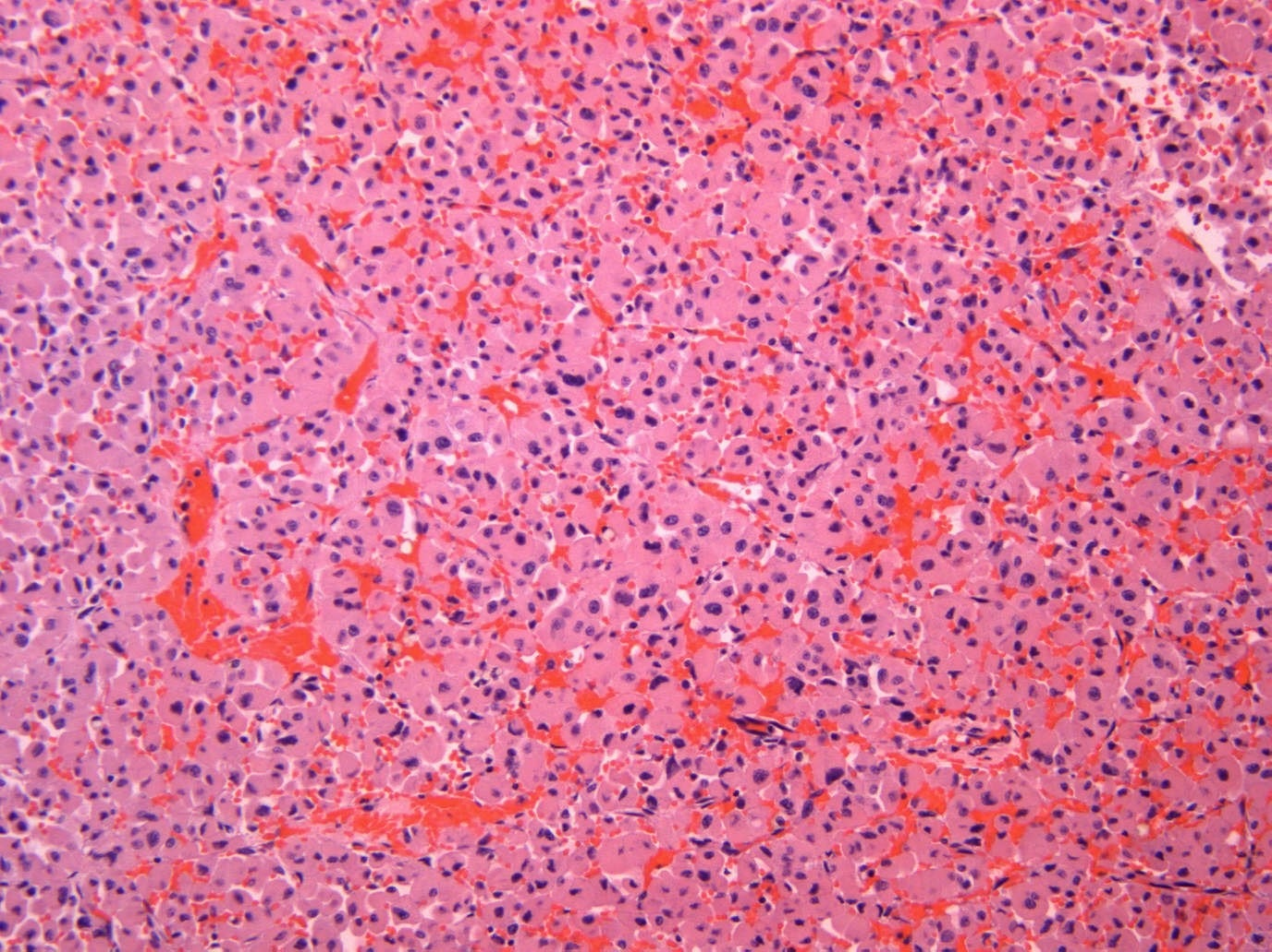
Oncocytic cells with abundant eosinophilic cytoplasm occurring in nests with a delicate fibrovascular network (H&E).

**Figure 5:**
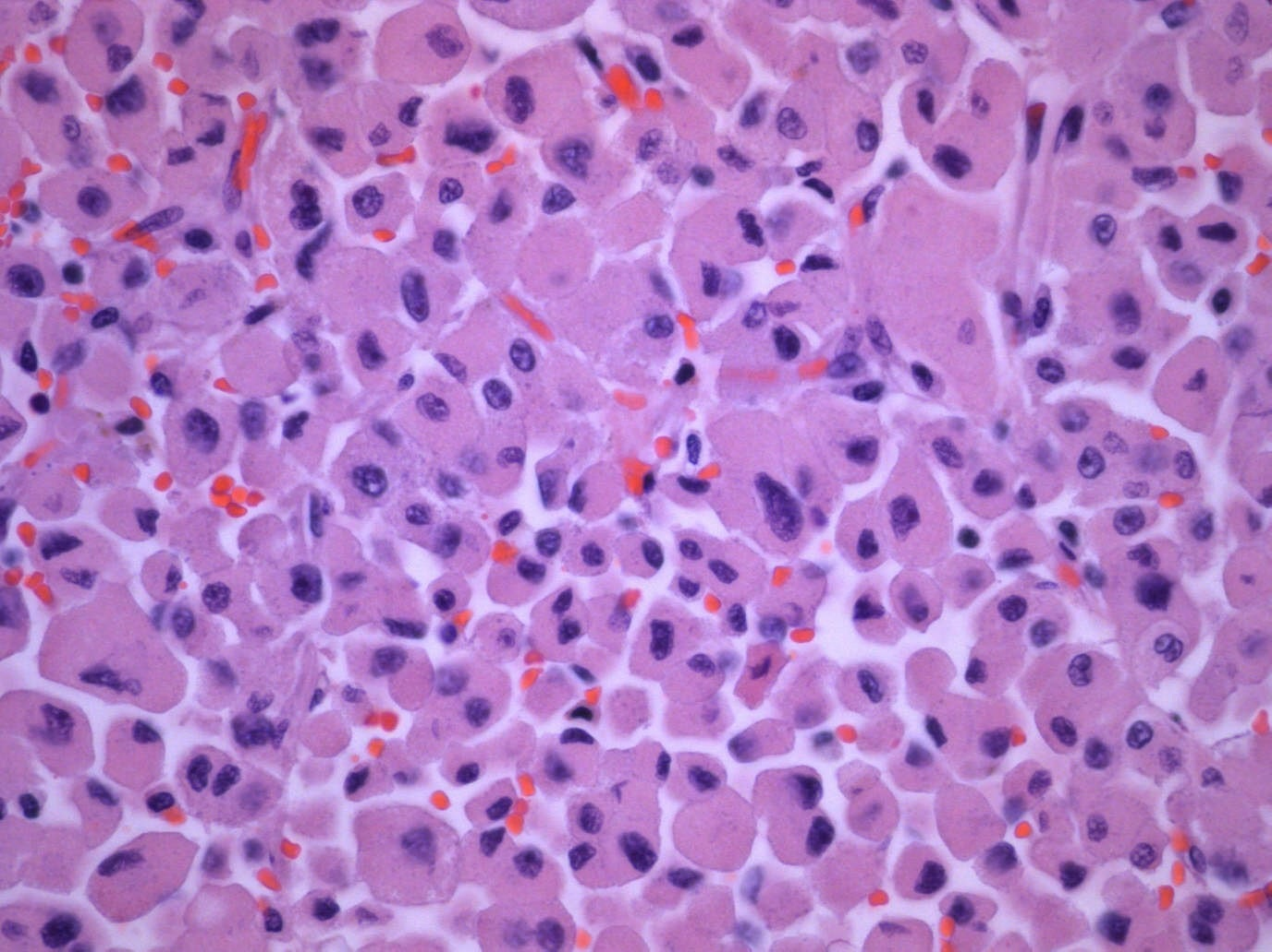
Oncocytic cells with significant cytologic atypia (HPF).

**Figure 6:**
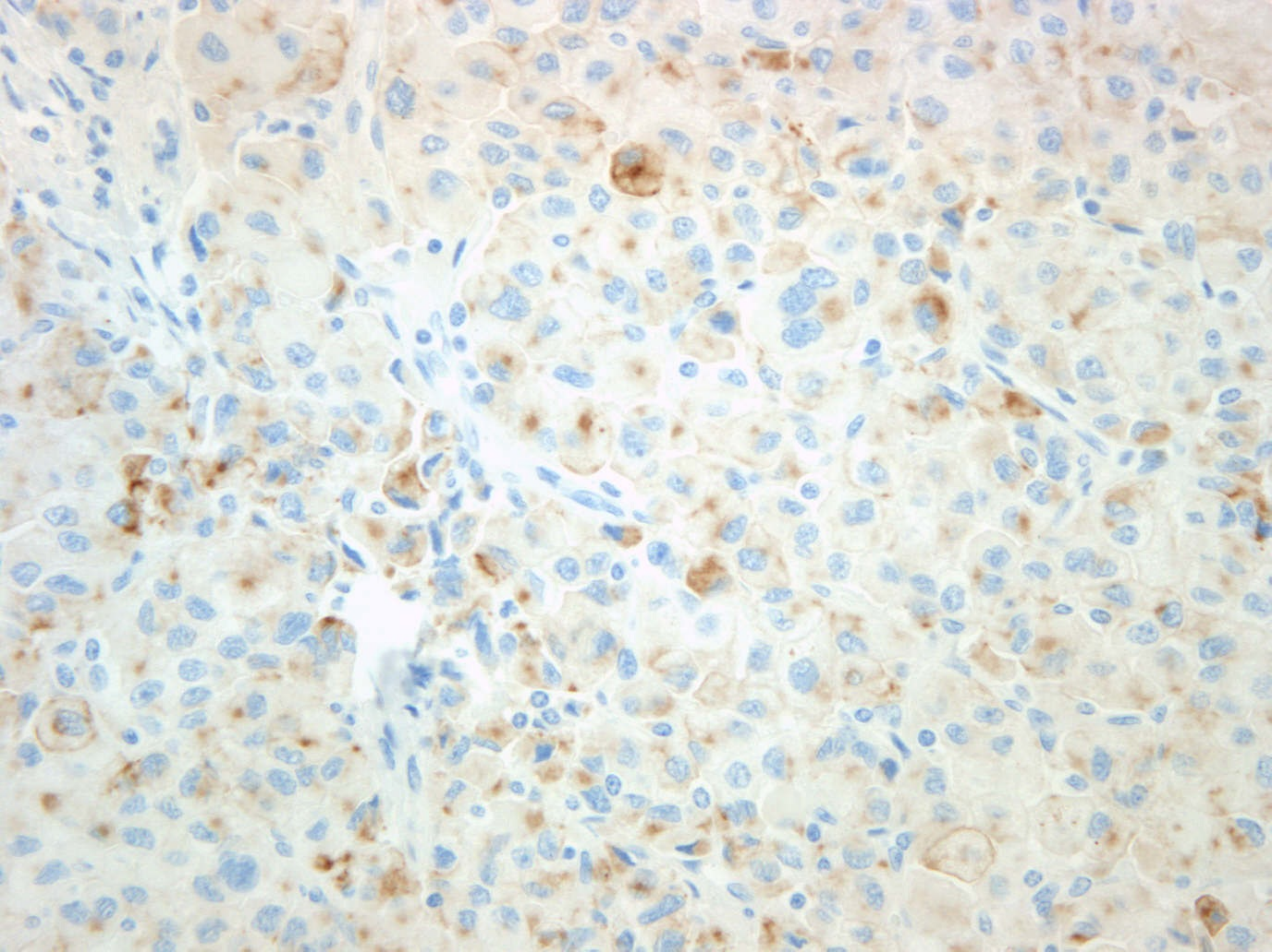
Oncocytic cells are focally positive for cytokeratin immunostain.

The specimen was sent to be reviewed at another laboratory. The pathology report noted that the sample consisted of a circumscribed adrenal tumor comprised of 97% oncocytic cells. There were rare intermixed adipose tissues and focal chronic inflammation present. The tumor cells were arranged in nests and trabecular aggregates supported by a delicate fibrovascular network (Figures 4-5). While the tumor size was large and some cytologic atypia was present, increased mitotic activity, atypical mitotic figures, and invasive features were not identified. The differential diagnosis included an adrenal cortical neoplasm, epithelioid angiomyolipoma, and a sex cord tumor of Mullerian origin. Ancillary immunostains showed focal positivity for cytokeratin AE1/AE3, Melan-A, and TFE-3 (Figure 6). Given the combination of cytokeratin and Melan-A positivity, the findings were most consistent with an OAN. While the major diagnostic criteria for malignancy were not satisfied, the large size of the tumor was compatible with a tumor of borderline (uncertain) malignant potential according to the Lin-Weiss-Bisceglia criteria [5].

A follow-up thyroid ultrasound was performed and revealed a complex nodule within the isthmus measuring 1.2 x 1.2 cm in diameter. A fine needle aspiration showed abundant follicular cells and colloid, consistent with a benign follicular nodule. However, the hypermetabolic nodule persisted on the PET-CT scan done as a follow-up on the adrenal tumor. A total thyroidectomy was performed, and a 1 x 0.8 x 0.6 cm nodule was identified in the left lobe at the level of the isthmus. The pathology report noted that the nodule was tan-yellow and firm/gritty on sectioning. The capsule was not complete, and a positive inked margin was identified. Microscopically, there were papillary structures with the classical optically clear nuclei, consistent with papillary thyroid carcinoma.

## Discussion

Adrenocortical oncocytoma is a very rare abnormality observed within the adrenal cortex [9]. It is usually benign, non-functioning, and diagnosed incidentally or during an investigation for symptoms that were not attributable to the tumor, such as abdominal pain, hematuria, essential hypertension, episodic vomiting, ascites, and edema of the lower extremities [10]. OAN has no precise age distribution (median age at diagnosis: 46 years), with a female to male predominance (1.8:1) [6]. Most of the OAN are well circumscribed, capsulated, ranging in size from 3 to 15 cm in greatest dimension (average: 8.4 cm), and weigh from 30-865 g [4].

CT and MRI are accurate in identifying the adrenal gland as the source of benign OAN. However, they have little value in the assessment of the origin of malignant tumors. The inhomogeneous appearance, increased attenuation on CT images, and absence of loss of signal intensity on opposed-phase MRI exclude adrenal adenoma and are suggestive of OAN. CT and MRI findings cannot be used to differentiate benign and malignant oncocytic neoplasms based on the criteria used for distinguishing adrenal cortical adenoma from carcinoma. A size less than 4 cm usually suggests a benign adrenal tumor. However, the average size of benign OAN is 7.6 cm; therefore, the size criteria used for adenoma would not be reliable for benign oncocytic neoplasms [11-12]. In this case, the large size and inhomogeneous appearance of the mass were suspicious and enough to indicate further intervention. On PET scan, it is usually observed as a hypermetabolic adrenal gland mass resembling cancer, as in our case. This may be due to the numerous intracellular mitochondria and intense hypermetabolism of glucose [13]. Son, et al. reported a case of OAN with BMP that showed high FDG uptake on PET scan, and the tumor recurred four years after surgery. He stated that tumors with high FDG uptake should not be simply classified as a false positive tumor and might require long-term follow-up with clinical, hormonal, and imaging evaluations [7].

The preoperative diagnosis is often a non-functioning tumor of the adrenal gland incidentally found on imaging performed for indications other than adrenal disease. A biochemical work-up has to be completed before any surgical intervention to rule out the presence of a primary functioning mass like a pheochromocytoma. Image-guided adrenal biopsy (IGAB) is relatively safe, with a complication rate of 2.8%, including adrenal and liver hematoma, pancreatitis, pneumothorax, adrenal abscess formation, and, rarely, tumor recurrence along the needle track [14-15]. IGAB can distinguish between an adrenal tumor and a metastatic tumor. However, it cannot differentiate between a benign adrenal mass from the less common adrenal carcinoma. Therefore, fine needle aspiration (FNA) is usually recommended when a nonadrenal malignancy is suspected in the setting of a known tumor elsewhere or when infection is a possibility [16]. Other authors reported that IGAB remains useful in diagnosing or excluding potential adrenal adenomas in patients with indeterminate preoperative imaging characteristics [17-18]. In this case, FNA was interpreted as malignant with no definitive origin, and the procedure was uneventful. However, its indication, in this instance, is questionable, and a surgical resection might have been directly performed.

The approach to an adrenal mass depends on its size and function. Adrenalectomy is the standard since OAN usually present as a large adrenal mass, and the CT/ MRI features are not usually suggestive of an adenoma. A laparoscopic approach carries less morbidity and a quicker patient recovery when compared with open adrenalectomy. Nevertheless, safe laparoscopic resection of large or potentially malignant adrenal tumors remains a matter of scientific debate [2, 11]. A surgical resection of an OAN with the assistance of a robotic system has also been reported. While adjuvant therapy has been described for adrenocortical carcinoma, its use in oncocytic neoplasms is unclear [3]. Therefore, adjuvant therapy has to be individualized based on each case. In this case, the mass was uneventfully removed with an open laparotomy, and radiation therapy was offered to the patient since there was a positive margin. However, the patient declined.

In order to distinguish between benign and malignant OANs, a combination of histological features has been used. The Weiss system assesses a total of nine histologic features: Fuhrman nuclear grade, mitotic rate > 5 per 50 high-power field, atypical mitoses, clear cell composition less than 25% of the entire tumor mass, diffuse architecture, necrosis, venous invasion, sinusoidal invasion, and capsular invasion. Weiss found that tumors with fewer than two of these features never metastasized, whereas those with more than four typically reoccurred or metastasized [19]. The Weiss system was modified by Bisceglia, et al. in 2004, who divided the histologic features into major and minor ones [5]. According to these criteria, the presence of one major criterion (high mitotic activity, atypical mitoses, or venous invasion) indicates malignancy. The presence of one to four minor criteria (large size, necrosis, capsular, or sinusoidal invasion) implies uncertain (borderline) malignant potential. The absence of any of the criteria suggests a benign tumor. Duregon, et al. proposed to use the “reticulin algorithm” (RA) as a fast, easy to interpret system with high reproducibility to differentiate adrenocortical tumors. This technique is a two-step procedure (reticulin disruption evaluation, followed by the recognition of three Weiss criteria, namely, necrosis, mitotic count, and venous invasion) [20].

The histological hallmark of OANs is the predominance of oncocytes, epithelial cells characterized by abundant granular eosinophilic cytoplasm due to abnormal mitochondrial accumulation. There is a debate as to how many oncocytes are required for a tumor to be classified as oncocytic [6]. We, in agreement with most authors, used the definition proposed by Bisceglia, et al. in which tumors composed of more than 90% oncocytes were considered purely oncocytic, those with 50%-90% oncocytes were mixed, and those with less than 50% oncocytes were adrenocortical neoplasms with focal oncocytic differentiation [3]. Several authors proposed that an oncocytoma has to be confirmed ultrastructurally or by immunohistochemistry using antimitochondrial antibody mES-13 [5]. The immunohistochemical profile of OANs is similar to that of other adrenocortical neoplasms. OANs are typically positive for vimentin, synaptophysin, Melan-A, and inhibin-α and variably positive for cytokeratin antibodies. However, chromogranin-A, S-100 protein, HMB-45, and epithelial membrane antigen(EMA) are usually negative [5-6, 21]. In this case, the tumor was formed of 97% oncocytic cells. It was positive for cytokeratin and Melan-A, but negative for chromogranin-A, S-100 protein, and HMB-45, which is consistent with other authors.

The prognosis of OAN depends on its classification according to the LWB system [5]. This method was validated by Wong, et al. to distinguish OANs regarding their future risk of recurrence, metastasis, and death [6]. OAN of borderline (uncertain) malignant potential seems to have a relatively benign clinical behavior with a reported three recurrences out of 47 cases over a median follow-up of 96 months [6]. Son, et al. reported a recurrence four years after the resection of an OAN with BMP [7]. Recurrence has been described in patients with an adrenal oncocytic carcinoma up to seven years after the removal of an adrenal tumor [8]. There was no evidence of metastases in our patient after the mass was surgically removed. A follow-up PET scan was performed six months after the initial diagnosis and showed no residual adrenal mass or metastasis. The patient will be followed-up to check for any recurrence or metastases every six months for two years and then yearly for at least five years.

Papillary thyroid cancers (PTCs) frequently show an increase in mitochondrial content [22], which is a characteristic of oncocytic tumors, and an oncocytic variant of PTCs has been described in the literature [23]. The genetic mutation responsible for mitochondrial accumulation in oncocytic cells might be linked to tumorigenesis. However, the exact relation remains unclear [24].

## Conclusions

Oncocytic adrenocortical neoplasms should be considered in the differential diagnosis of adrenal masses. When an OAN is identified, it should be classified regarding its biological behavior as benign or malignant using the Lin-Weiss-Bisceglia system and eventually the reticulin algorithm of Duregon, et al. OANs can be confirmed ultrastructurally or by immunohistochemistry. Further studies are warranted to determine the length, frequency, and parameters needed to follow-up on patients with oncocytic neoplasms of borderline or uncertain malignant potential. Studying the gene mutations in patients presenting with oncocytic malignancies and other tumors that demonstrate mitochondrial proliferation as papillary thyroid cancer in this report might help to better understand the role of mitochondrial proliferation in cancer development.
